# Genome-wide analysis of structural variants reveals genetic differences in Chinese pigs

**DOI:** 10.1371/journal.pone.0186721

**Published:** 2017-10-24

**Authors:** Ruifei Yang, Suyun Fang, Jing Wang, Chunyuan Zhang, Ran Zhang, Di Liu, Yiqiang Zhao, Xiaoxiang Hu, Ning Li

**Affiliations:** 1 Beijing Advanced Innovation Center for Food Nutrition and Human Health, China Agricultural University, Beijing, P. R. China; 2 State Key Laboratory of Agrobiotechnology, College of Biological Sciences, China Agricultural University, Beijing, P. R. China; 3 Institute of Animal Industry, Heilongjiang Academy of Agricultural Sciences, Harbin, P. R. China; 4 National Engineering Laboratory for Animal Breeding, China Agricultural University, Beijing, P. R. China; University of Queensland, AUSTRALIA

## Abstract

Pigs have experienced long-term selections, resulting in dramatic phenotypic changes. Structural variants (SVs) are reported to exert extensive impacts on phenotypic changes. We built a high resolution and informative SV map based on high-depth sequencing data from 66 Chinese domestic and wild pigs. We inferred the SV formation mechanisms in the pig genome and used SVs as materials to perform a population-level analysis. We detected the selection signals on chromosome X for northern Chinese domestic pigs, as well as the differentiated loci across the whole genome. Analysis showed that these loci differ between southern and northern Chinese domestic pigs. Our results based on SVs provide new insights into genetic differences in Chinese pigs.

## Introduction

Structural variants (SVs), including deletions, duplications, and inversions, widely exist in the genome. It has been estimated that the occurrence rate of deletions is 0.113 deletions per haploid genome per generation in humans [1,2]. Current advances in genome sequencing techniques have enabled the high-throughput and accurate detection of SVs. For example, the recent releases of a high-resolution deletion map of 1,092 human genomes which contains 8,943 high-quality deletions and an SV map of 2,504 individuals provided a comprehensive survey of 68,818 SVs in the human genome [2,3].

It has been widely reported that SVs cause various phenotype changes. For example, a 900-kb inversion polymorphism enriched in the European population was discovered to be associated with increased fecundity in humans [4]. In animals, an inversion on chicken chromosome 7 causes the transient ectopic expression of *MNR2*, resulting in the rose-comb phenotype [5]. The white coat color in goats and pigs was found to be caused by duplications of *ASIP* [6] and *KIT* [7,8], respectively.

Pigs were domesticated independently in East Anatolia and China approximately 10,000 years ago [9]. During long-term domestication, human activities have affected numerous phenotypes, such as ear shape, body composition, and growth traits [10–12]. Compared to wild boars, domestic pigs exhibit much better production and reproduction performance, owing to human-mediated selections [12]. In addition, animals domesticated in various areas also achieved successful adaptation to distinct environments [13,14]. For example, Chinese indigenous domestic breeds distributed in the vast geographical region of China, where the environmental temperatures are extremely discrepant [10].

Restricted by technical limitations, previous studies on pig genomic changes mostly focused on single nucleotide polymorphisms (SNPs) and unbalanced SVs, which are also called copy number variants (CNVs) [15–18]. Although some SVs are known to affect phenotype severely, current studies on genome-wide large-scale variations, including deletions, inversions or tandem duplications, are very limited. A recent study on SVs of 252 pigs from the Chinese Taihu area identified associations between SVs and disease resistance, as well as docile temperament, suggesting the importance of SVs with respect to domestication [19]. Zhao et al. characterized genome-wide SVs in 13 Chinese and European pigs, and found a Chinese pig-specific SV region spanning 35 Mb on chromosome X that evolved at different rates between Chinese and European pigs [20]. Even with efforts from previous studies, however, the selection and population differentiation of SVs among Chinese indigenous breeds remain largely unclear.

In this study, we performed a comprehensive analysis on SVs of Chinese pig breeds. We detected selection signals and identified genome-wide candidate loci for local adaptation or domestication using an SV catalog. Our results reveal genetic differences on SVs in Chinese pigs, which further potentially affect phenotypic changes and local adaptation differences.

## Results

### Building SV sets

Whole genome re-sequencing data of 66 Chinese pigs were downloaded from Ai’s study [13]. Reads from each individual were mapped to the pig reference genome (Sscrofa10.2) using BWA with default settings. For each individual, we calculated the genome coverage and sequencing depth from the bam files. All individuals have a minimum sequencing depth of twenty-fold and a genome coverage over 88%, and it is thus sufficient to identify precise SVs (Table 1). Five different tools were applied to identify SVs simultaneously. Due to the limitation of each tool and our research objective, we only included deletions, inversions, and tandem duplications. For each individual, an SV was called if the evidence was supported by at least two tools. Altogether, we identified 66,921 SVs with sizes varying from 50 to 10,000,000 bp, including 59,138 deletions, 4,938 inversions, and 2,845 tandem duplications (Table 2 and S1 Table). We created a genotyped set of SVs with the accurate breakpoints assigned by DELLY based on the merged set, and thus genotype information of each SV was obtained (Table 2 and S2 Table). We also created a precise set of SVs of which SVs were less than 60 kb from the merged set, and with the breakpoints refined at single-nucleotide resolution via local *de novo* assembly (Table 2 and S3 Table). It is worth noting that the inferred breakpoints of inversions and tandem duplications would be less accurate than those of deletions due to their complexity and the limitation of the local *de novo* assembly method [2], thus leaving deletions for further analysis which were derived from the precise set.

**Table 1:** Statistics of mapping depth, coverage, and all types of SVs.

Pig breeds	Number	Average sequencing depth	Average sequencing coverage (%)	Structure variants		
				Deletion	Inversion	Tandem duplication
Min	6	26.3	88.4	28,415	2,919	870
Erhualian	2	27.0	88.4	22,590	2,343	666
Hetao	6	24.7	88.1	30,113	2,913	894
Laiwu	6	26.8	88.2	28,325	2,910	894
Luchuan	6	25.8	88.4	28,404	2,722	897
Bamaxiang	6	27.2	88.4	31,284	2,950	1,045
Wuzhishan	6	26.5	88.5	32,583	2,953	1,026
Tibetan (Gansu)	4	26.0	88.3	26,296	2,579	766
Tibetan (Sichuan)	6	27.3	88.5	31,992	2,977	1,017
Tibetan (Tibet)	6	24.2	88.5	32,358	2,984	951
Tibetan (Yunnan)	6	26.7	88.6	32,550	2,990	1,007
Wild	6	23.7	88.3	34,085	3.004	944

**Table 2:** Statistics of information of all types of SVs.

SV class	Merged set	Precise set^a^	Genotyped set	Average number of SV sites per individual	Median size of SV sites (bp)	Median size covered per individual (kb)	Median breakpoint precision (start position/end position bp)	Median allele counts per individual
Deletion	59,138	33,628^b^	28,685	17,378	311	109,095	6/2	24,049
Inversion	4,938	547	1,346	2,009	34,028	179,122	33/41	1,961
Tandem duplication	2,845	534	656	470	4,929	41,449	40/46.5	402

^a^SVs < 60 kb

^b^56.86% of deletions have precise breakpoints.

### NHR and TEI are important SV formation mechanisms in Chinese pig genome

**Fig 1:**
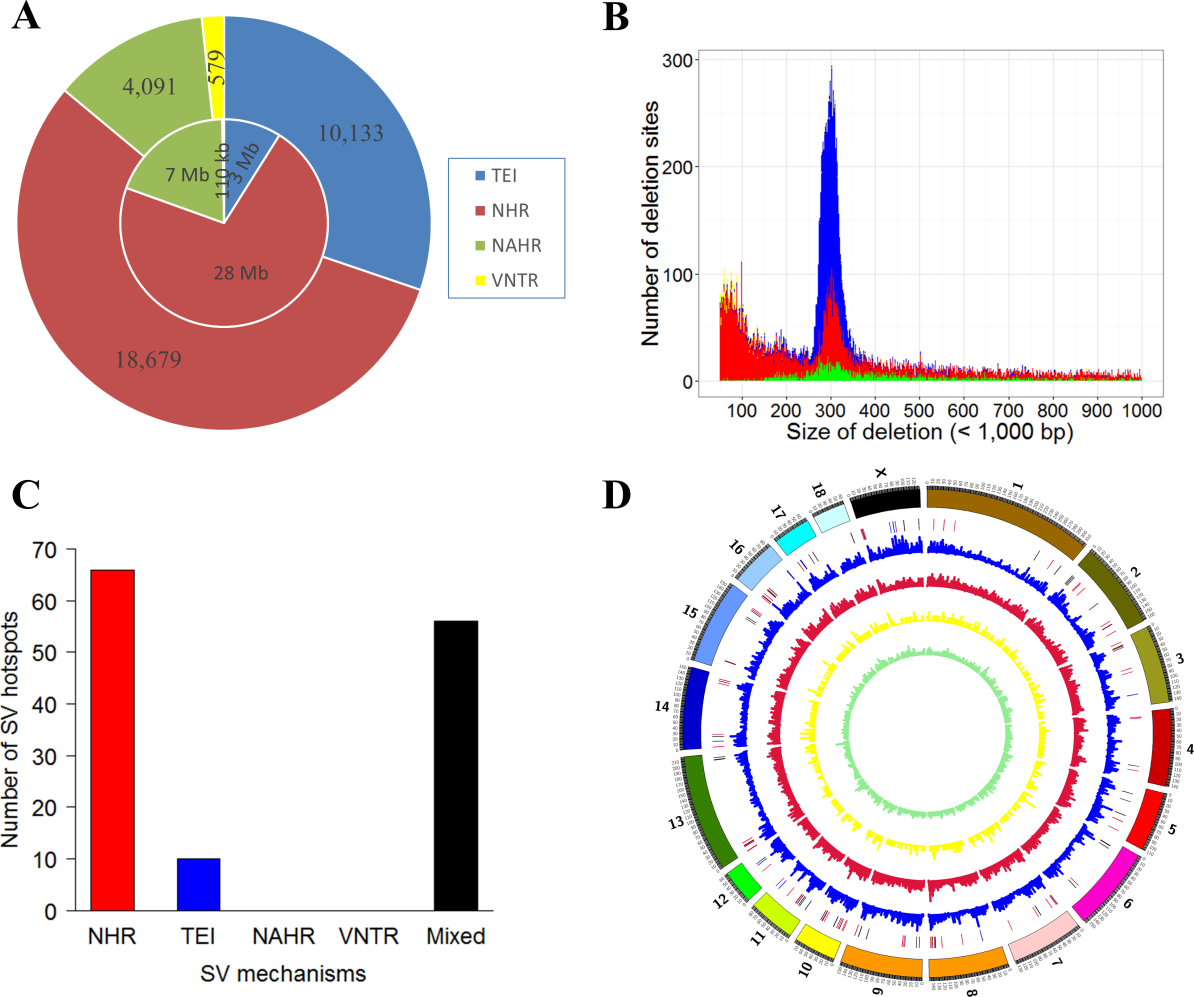
SV formation mechanisms of 66 Chinese pigs. (A) Pie chart of formation mechanisms (33,482 SVs) of recalibrated deletions. The inner and outside circles represent the lengths and occurrence numbers of all recalibrated SVs, respectively. (B) Length distribution of different forms of deletions. Color reference is same as in A. (C) Formation mechanisms of deletions in SV hotspots. (D) Distribution of deletions generated by different formation mechanisms in the whole genome. The highlight red, blue and black regions represent hotspot of NHR, TEI and mixed distributions, respectively. Color reference of the inner circles is same as in A.

According to previous studies, the mechanisms of SV formation include: 1) nonallelic homologous recombination (NAHR), which is caused by long stretches of similar sequences at the flank of breakpoints [21]; 2) nonhomologous recombination (NHR) [22], which is generated in the nonhomologous region involving nonhomologous end joining (NHEJ) [23,24], stalling and template switching (FoSTeS), or microhomology-mediated break-induced repair (MMBIR) [25]; 3) transposable element insertions (TEI) [26]; and 4) expansion or contraction of variable numbers of tandem repeats (VNTR) [27]. Using the BreakSeq analysis pipeline [28], we studied the formation mechanisms of all 33,628 deletions in the precise set (S4 Table). In total, the formation mechanisms of 33,482 deletions were successfully inferred (Fig 1A). NHR was found to be the major formation mechanism of deletions, determined either by SV count (55.79%) or total SV length (73.47%). In addition, we determined that TEI accounted for overall 30.26% deletion events or 7.87% of the total deletion length (Fig 1A). The NAHR and VNTR mechanisms, which accounted for 12.22% and 1.73% in event percentages or 18.37% and 0.29% in length, were the next prevalent mechanisms.

As shown in Fig 1A, since we observed a disproportion of total counts and total length for TEI, we further examined the length distribution of all deletions. Consistent with previous reports in humans and pigs [2,13,20], there was an evident peak around 300 bp, as shown by the length distribution of all deletions (S1 Fig). Further analysis of the mechanisms of SV formation confirmed that TEI SVs were enriched around the 300 bp peak (Fig 1B). Since 300 bp is the approximate length of *SINE/Alu* elements, this result agreed with previous reports that *SINE/Alu* elements were the most representative TEI in pigs [11,29,30].

In addition to prevalence, we are also interested in mechanisms that drove SV clustering in the pig genome. We performed a runs test for randomness on all of the deletions in the pig genome. Results demonstrated that the deletions were non-randomly distributed across the genome (Z value = -243.177, p < 0.001). We thus searched for SV hotspots in the genome (25 SVs per 1 Mb region as the outlier threshold according to the 95 percentile of SV counts for all windows) using a non-overlapping sliding window of 1 Mb. We further defined a single-mechanism-formation SV hotspot if more than 50% of SVs formed by a single mechanism in this region. In all, we found 132 SV hotspot regions, with 66 regions dominated by NHR and 10 regions dominated by TEI (p < 1e-6, Fisher’s test) (S5 Table). NHR was the most dominant formation mechanism compared to others (p < 0.001, ANNOVA test) (Fig 1C and 1D and S2 Fig).

### Effective population analysis of SVs

**Fig 2:**
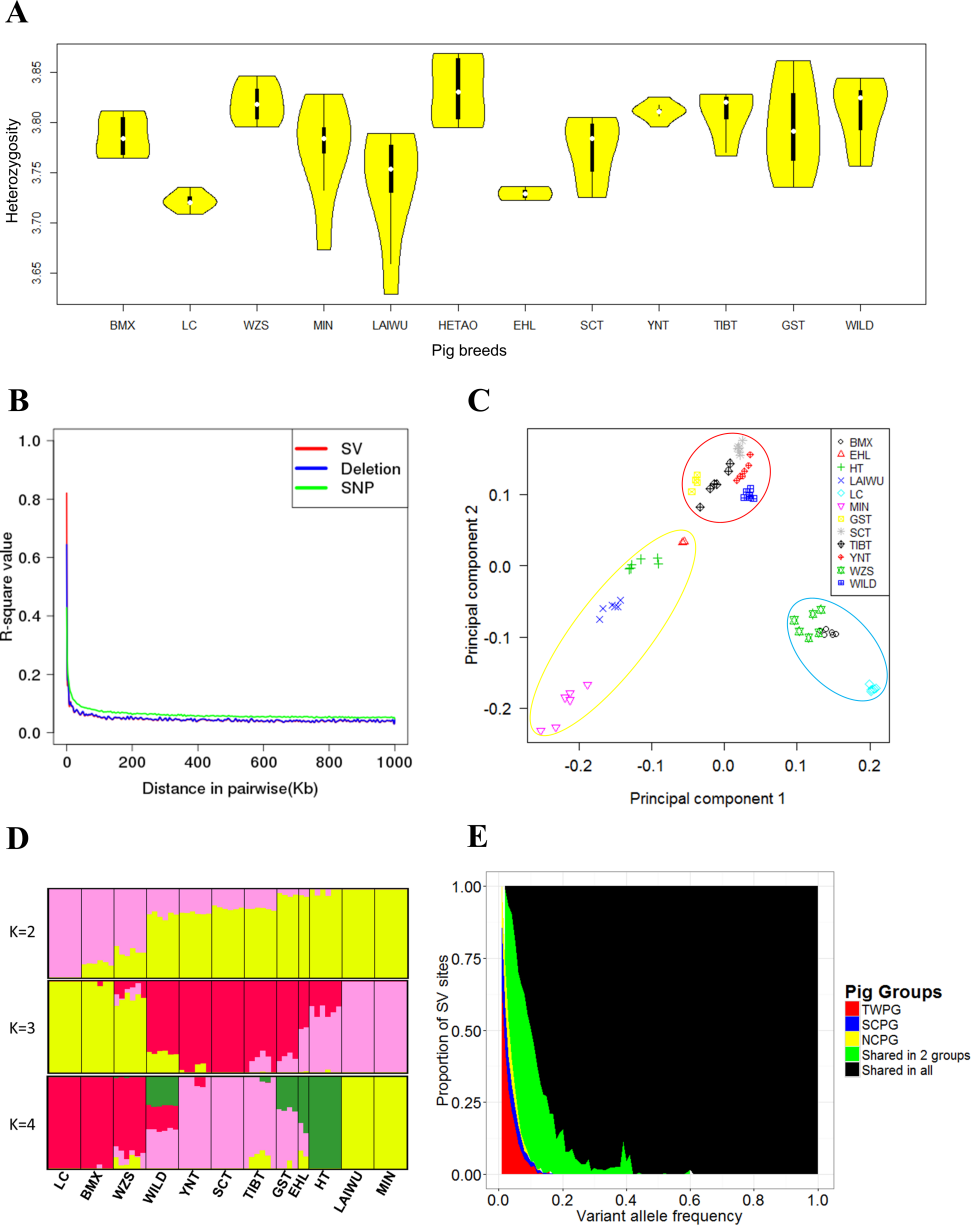
Population genetic properties in distinct regions of Chinese pigs. BMX, Bamaxiang; EHL, Erhualian; HT, Hetao; LAIWU, Laiwu; LC, Luchuan; MIN, Min; GST, Tibetan (Gansu); SCT, Tibetan (Sichuan); TIBT, Tibetan (Tibet); YNT, Tibetan (Yunnan); WZS, Wuzhishan; WILD, Southern Chinese wild boar. (A) Log10 values of deletions heterozytosity of all pig breeds. (B) The LD decay of SVs, deletions, and SNPs. (C) Principal component analysis based on deletions of 66 Chinese pigs. Yellow, red, and blue circles stand for NCPG, TWPG, and SCPG, respectively. (D) Admixture analysis of deletions in Chinese pigs (K = 2, 3, and 4). (E) Frequency distribution of SVs in different groups of Chinese pigs, in which the red, blue, and yellow bars stand for specific SVs in TWPG, SCPG, and NCPG, respectively. The green bars represent SVs shared by any two groups of Chinese pigs, and black bars denote SVs shared by all three groups.

Although SVs are widely distributed, as shown in Fig 1D, the numbers of SVs are lower by two orders of magnitude than the numbers of SNPs, which might challenge the practicability for the population-level analysis. To address this question, we firstly plotted the heterozygosity distributions of deletions and SNPs for each breed. As shown in Fig 2A and S3 Fig, the two plots were highly similar, showing that the SVs obtained in this study were quite informative. Unfortunately, the heterozygosity distributions of inversions and tandem duplication seemed to be different from those of deletions (S4 Fig), which might be due to insufficient counts. As a common approach for measuring genetic diversity and determining the number of markers needed for population analysis, we next compared the pattern of linkage disequilibrium (LD) decay for SVs and SNPs for the whole population. As we can observe in Fig 2B, the patterns are, again, very similar for SNPs and SVs. Principal component analysis (PCA) is one of the most popular methods for population structure analysis. We carried out a PCA using the deletions in the genotyped set. As shown in Fig 2C, the Chinese pig population was divided into three groups: 1) the southern Chinese domestic pig group (SCPG) (Wuzhishan, Luchuan, and Bamaxiang pigs); 2) the northern Chinese domestic pig group (NCPG) (Min, Laiwu, Hetao, and Erhualian pigs); and 3) a third mixed group including four regions of Tibetan pigs (Gansu, Sichuan, Tibetan, and Yunnan) and wild boars (TWPG). A basically similar result was found when using the inversions and tandem duplications for the analysis (S5 Fig). Agreeing with PCA analysis, similar patterns were observed from the population stratification analysis using Admixture software (Fig 2D, S6 and S7 Figs), and these findings were consistent with previous study [13]. Finally, we checked SVs that specifically belonged to, or were shared by, different groups. As shown in Fig 2E, TWPG contains more specific SV allele sites than the other two groups (TWPG: 98.25 per genome, SCPG: 57.17 per genome, and NCPG: 42.50 per genome). The distribution frequencies of deletions, inversions, and tandem duplications were similar (S8–S10 Figs), and they were all consistent with the higher genetic diversity of Tibetan pigs and wild boars compared to domestic pigs [11,30]. We again repeated the corresponding analysis using SNPs, and the results were virtually identical (S11 Fig).

### Selection on deletions on chromosome X in NCPG

**Fig 3:**
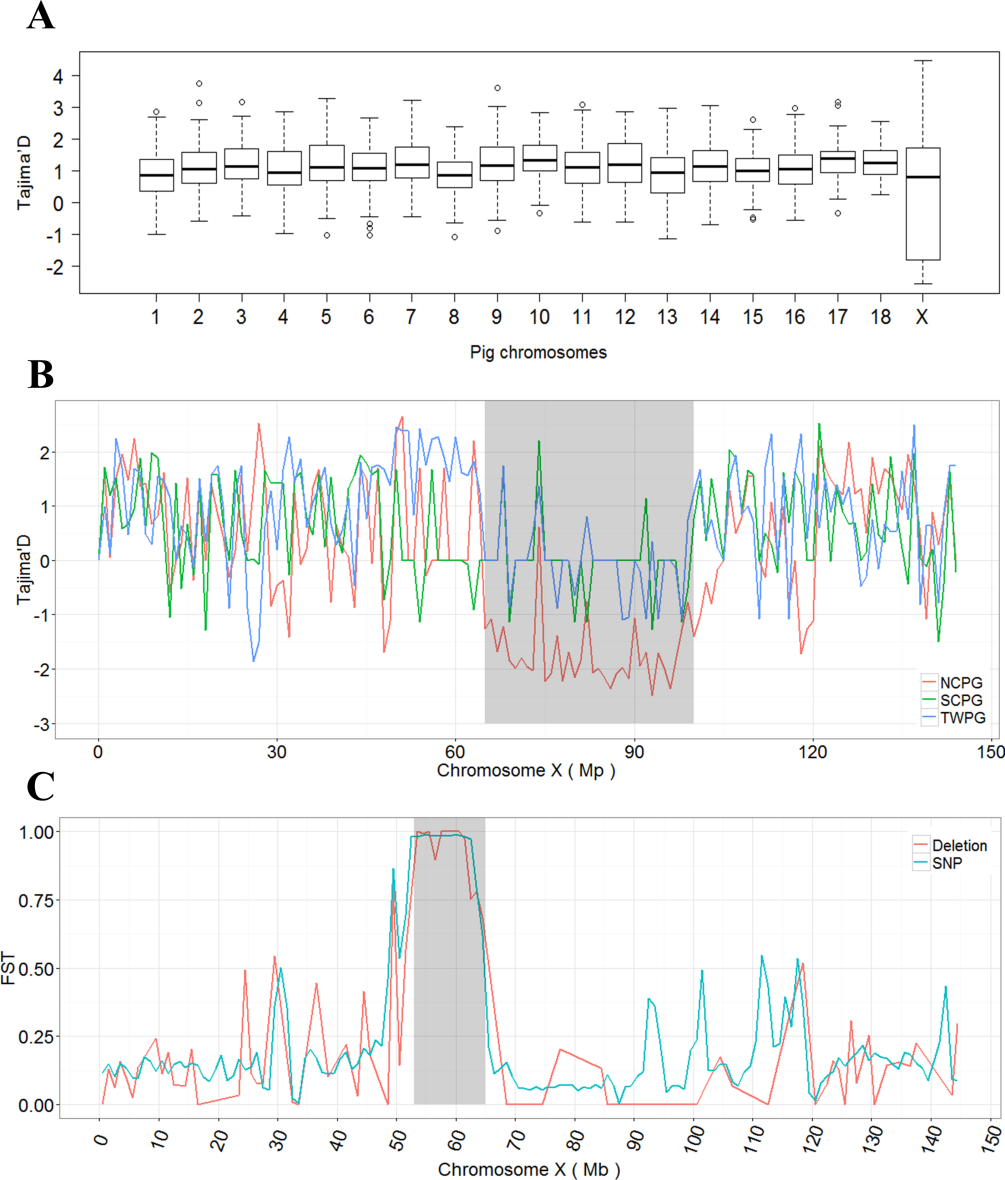
Tajima'D value and FST show a selective sweep of deletion on chromosome X in Chinese pigs. (A) Tajima’D values of deletions on different chromosomes. (B) Tajima’D values of deletions on chromosome X in NCPG, SCPG, and TWPG groups. The gray region represents the Chinese pig-specific SV region (65 to 100 Mb region on chromosome X), where the NCPG shows a continuous negative status. (C) Fst values of deletions and SNPs in the comparison of NCPG and SCPG. The gray region stands for the most significantly differentiated block (53 Mb-65 Mb on chromosome X) between the NCPG and SCPG.

Due to the insufficient number and more complex breakpoints of inversions and tandem duplications, we only included deletions for further analysis. Using a sliding window approach, we calculated Tajima’D on the deletions for all 66 samples. As shown in Fig 3A, the distributions of Tajima’D value across chromosomes were comparable, except chromosome X. Tajima’D on chromosome X spanned towards a negative value with a significantly larger range compared with other chromosomes (p < 0.001, ANNOVA test). By calculating the values of each group separately, we confirmed that the exceptionally high Tajima’D variations on chromosome X were introduced by NCPG (S12 Fig) and further decreased to the 65 to 100 Mb region on chromosome X with an unusually low Tajima’D (Fig 3B). Interestingly, this is the same region recently identified as a specific hotspot of SVs in the Chinese population [20]. Consistent with this, a very low Tajima’D value of the same 35 Mb region was also observed in the NCPG by analyzing the SNPs (S13 Fig), confirming a strong selective sweep for NCPG at this region.

To further investigate the selection signals on chromosome X, we calculated Fst for the SCPG vs. NCPG group based on the deletions in the genotyped set. The most differentiated region was found to be from 53 to 65 Mb, which comprises 75 genes (S6 Table) between NCPG and SCPG. We also detected the signal of SNPs, resulting in the same large differentiated region as deletions (Fig 3C). We further examined the most differential SNPs (Fst > 0.8 in SCPG vs. NCPG comparison) in an additional 26 European pigs obtained from NCBI (16 Duroc, seven Landrace, and three Yorkshire pigs) (S7 Table). Seventy-five missense mutations for 26 genes were identified (S8 Table). Among these 75 SNPs, all were fixed in the Chinese indigenous breeds (SCPG or NCPG), but hardly in the European group, which indicated specific selection signals in the Chinese pigs. Interestingly, a previous study demonstrated that differences in this region were the result of an introgression from a distant Sus species for the NCPGs [13].

### Genetic differences on autosomes among Chinese pigs

As shown above, the differences between SCPG and NCPG on chromosome X were most pronounced. There could be large amounts of differentiated loci on autosomes. Thus, we extended our analysis to the whole pig genome (excluding chromosome X, Y, and MT, and unplaced scaffolds). After calculating Fst on the autosome-genome deletions and selecting those that surpassed the top 5% significant Fst level (p < 0.001, permutation test), there remained a total of 638 and 667 differentiated loci in TPG (Tibetan pigs from four regions of China) vs. DPG (SCPG and NCPG mixed domestic group) (Fst > 0.206) and SCPG vs. NCPG groups (Fst > 0.391).

For 638 differentiated deletion loci in TPG vs. DPG (S14 Fig and S9 Table) comparison, GO analysis was performed on the associated genes where the loci overlapped with the transcript region, or within 200 kb distance to the nearest transcript starting sites (TSS). Result showed functions related to plateau adaptation or domestication traits (S10 Table), such as digestive system development (p = 0.00017), nervous system development (p = 0.00062), response to X-ray (p = 0.00076), positive regulation of developmental process (0.00037), skeletal system morphogenesis (0.00889), and reproductive system development (0.02928).

We investigated the differentiated loci between SCPG and NCPG as well (S11 Table), GO enrichment analysis showed over-representation of functional categories related to the local adaptation to hot and cold temperatures in low- and high-latitude areas (S14 Fig and Table 3), including sprouting angiogenesis (*TEK*, *KLF4*, *EFNB2*, *E2F8* and *PARVA*; p = 0.0031), cardiac septum development (*SMAD6*, *SAV1*, *PITX2*, *ADAMTS6* and *PARVA*; p = 0.0136), kidney development enriched category (*SMAD6*, *EFNB2*, *ARID5B*, *ADAMTS6*, *WT1*, *KLHL3*, *CALB1*, *TFAP2A*, *PKD2* and *CENPF*; p = 0.0053), and transmission of nerve impulse (*GRM7*, *GLRA1*, *SCN3A* and *SCN8A*).

**Table 3:** GO enrichment of genes which are identified in the SCPG vs. NCPG comparison to be affected by the top 5% of significantly differentiated deletion sites.

GO ID	GO term	Gene number	P value
GO:0002040	Sprouting angiogenesis	5	0.0031
GO:0001822	Kidney development	10	0.0053
GO:0045453	Bone resorption	4	0.0069
GO:0003279	Fat cell differentiation	5	0.0136
GO:0019226	Transmission of nerve impulse	4	0.0235
GO:0035904	Aorta development	3	0.0347
GO:0007517	Muscle organ development	9	0.0413

A previous study demonstrated similar gene functions in the differences between SCPG and NCPG [13]. For further exploration of these differences, we employed LD analysis for these deletions sites with nearby SNPs. In the 667 deletions sites, we only found that seven sites were in lower LD with SNPs (r^2^ < 0.6), indicating their specific contribution to the genomic differences between SCPG and NCPG. One of the highest differentiated deletion sites (Fst = 0.511) occurred in the intron region of *FANK1*, which is testis-specific expressed and involved in the progress of spermiogenesis. Among all differentiated deletion sites, Chi-square test showed that more significantly differentiated sites were in higher LD with nearby SNPs (χ^2^ = 167.39, p < 0.001) compared to the sites that were less significantly differentiated. Furthermore, we performed Fst computation for genome-wide SNPs (excluding chromosome X, Y and MT, and unplaced scaffolds), and identified the top 5% significant sites (p < 0.001, permutation test), leaving 603,706 sites (Fst > 0.448). Among these sites, 7,526 SNP sites with high Fst level were in high LD (r^2^ > 0.9) with 356 deletion sites. Thus, we merged the overlapped regions which were either in high LD (r^2^ > 0.9) with differentiated deletions or differentiated SNPs, and obtained 341 clusters as the deletion anchored differentiated cluster (DEL-DC) (S12 Table). These clusters contain most differentiated regions between SCPG and NCPG, with the sizes ranged from 69 bp to 827,379 bp and summed up to ~8.43 Mb of the autosomes.

## Discussion

In this study, we generated a comprehensive SV map using high-coverage genome sequencing data of 66 Chinese pigs. We inferred SV formation mechanisms, and found that NHR and TEI constituted the major SV formation mechanisms and NHR formed SV hotspots mainly in Chinese pigs. Moreover, we confirmed the practicability of SVs for the population-level analysis. Although SVs occurred at a much lower frequency than SNPs, they still provided a good resolution for genome-wide analysis even within the evolutionarily close Chinese breeds. A series of population-level properties, including heterozygosity, LD decay, PCA, population stratification, as well as frequency distribution, all indicated that SVs were as informative as SNPs. Among the SVs, some false-positives might exist. A previous study had illustrated the low coverage and low quality regions (LQLC) of Sscrofa10.2 reference genome may cause false discovery of variants, especially for CNVs (86.3% of calls from previous studies were located in these regions) [31]. In our study, we found 43.26% of deletions were lying around the LQLC regions, which was largely lower than previous studies. In the genotyped set, we detected the a large amount of homozygous fixed SV variants (2,375 deletions, 806 inversions and 94 tandem duplications in the whole genome) occurred in these regions (1,782 deletions, 806 inversions and 94 tandem duplications in the LQLC regions), so these SVs were excluded in our further analysis.

We discovered a long-range selection signal on chromosome X, which agreed with a previous report that this region was under adaptive introgression and selected in the NCPG [13]. In addition, 26 genes were identified between the SCPG and NCPG in this region (S6 Table), which are promising key genes underlying the genetic differences in Chinese domestic pigs that are distributed in the south and north. For example, *AWAT2* participates in the synthesis of wax esters, which are important components of sebaceous gland lipids to prevent water evaporation and skin desiccation [32,33]; *P2RY4* facilitates the regulation of physiological activities, including blood pressure, renal sodium excretion, and neuron differentiation [34,35]. The two genes showed possible distinct local adaptation between the SCPG and NCPG. However, the regions detected by Tajima’D and Fst methods were not overlapping, this may be due to strategic differences in detecting signals between these two methods [36,37].

When extending to the autosomes, we first investigated differences between TPG and DPG, and the result was in agreement with previous findings [16,30,38], which indicated that SVs could also represent different genetic backgrounds of different Chinese pig populations. Thus, we further detected genetic differences based on SVs between SCPG and NCPG, the result showed local environmental adaptation related differences between these two groups, including *SMAD6*, *ADAMTS6* and *PKD2* affecting blood circulation and heat loss [39–43]; *PKD2*, *ENFB2* and *WT1* associated with kidney development [44–46], and it has been reported that the kidney weight tends to differ between in the cold and in the heat in several species [47]; *TFAP2A* affecting hair follicle differentiation [48]; and *GRM7*, *SCN3A*, *SCN8A* and *GLRA1* involved in transmission of nerve impulse [49–52], which agreed with the fact that the nervous system plays a key role in the first-line response of heat loss or production [53]. Further, we used SVs to define DEL-DC, which represented a hotspot region of variants located in high LD level. Several genes were found to be potentially related to phenotype differences between SCPG and NCPG, such as *IFT74*, *TPD52L3*, *PAFAH1B2* and *MTMR2*, which were found to be associated with spermatogenesis, and dominantly reproductive differences existed between these two groups [10]. *MEMO1* and *TAGLN* were involved in aorta development, suggesting differences in the regulation of blood circulation that affect thermoregulation [42,43]. However, we identified differentiated deletion sites mainly distributed in non-coding regions (S14 Fig and S12 Table), especially in the intergenic regions, suggesting important contribution of regulatory elements in the genetic differences between SCPG and NCPG. Deletions were grouped into three groups: 1) 288 deletions distant to TSS (> 50 kb, D50 group); 2) 171 deletions close to TSS (< = 50 kb, C50 group); and 3) 207 deletions located in intron regions (INTRON group) (S10 Table). We compared Fst statistics for each domestic breed (NCPG: Laiwu, Min, Hetao; SCPG: Bmx, Wzs, Luchuan) with six wild boars (WILD) for the three groups (S13 Table). We found that Fst of deletions in DEL-DC were significantly higher than those were not in DEL-DC for all three groups (p < 0.001, permutation test) (S15 Fig). Moreover, within DEL-DC, significant differences in Fst for deletion sites were observed in both D50 and C50 groups when comparing the differences between each breed of SCPG and wild boars and the differences between each breed of NCPG and wild boars (p < 0.001, permutation test) (S15 Fig). Further, we confirmed that the variances of Fst values between each breed of SCPG vs. WILD and each breed of NCPG vs. WILD in D50 group were much higher than that in C50 group (D value, see Materials and Methods) (p = 0.0377, permutation test). Therefore, it suggests that large differences existed in long distance regulatory elements between SCPG and NCPG. We performed GO enrichment analysis on the affected genes. The top 15 significant GO terms were almost enriched in neuron development-related categories (S14 Table), which suggests that human-mediated selections varied on SCPG and NCPG, and further affected domesticated differences between these two groups.

Altogether, the results of the present study revealed the important functionality of SVs to genetic differences, which further reflected phenotypic changes and local adaptation in Chinese pigs. These findings may provide new insights into differences in domesticated progress in Chinese pigs.

## Materials and methods

### Genome sequencing data accessing, mapping, and SNP calling

Illumina pair-end sequencing data analyzed for this study can be obtained from the NCBI Sequence Reads Archive, which was released by Ai and colleagues in a previous study [13] (SRA096093, http://www.ncbi.nlm.nih.gov/). Three Erhualian pigs were removed from this analysis because of low coverage. The final data comprise 20 northern domestic Chinese pigs (NCPG), 18 southern domestic Chinese pigs (SCPG), 22 Tibetan pigs from four regions of China (Gansu, Sichuan, Yunnan, and Tibet), and six wild boars (TWPG). Pig reference genome sequences were downloaded from ENSEMBL (ftp://ftp.ensembl.org/pub/release-82/fasta/sus_scrofa/dna/). We aligned the raw reads to the pig reference genome using BWA v0.7.10 [54] software. GATK v3.2.2 [55] was used to call SNPs. Variants with confidence/quality by depth (QD) below 20.0 and mapping quality (MQ) below 30.0 were removed.

### SV detection

DELLY v0.7.2 [56], Breakdancer v1.1.2 [57], Pindel v0.2.4 [58], CNVnator v0.3 [59], and Lumpy v0.2.12 [60] were used to discover SVs ranging from 50 bp to 10,000,000 bp for each sample, including deletions, tandem duplications, and inversions. Deletion detection was determined by merged results from all five methods, whereas detection of inversions was merged by the results of Breakdancer, Pindel, Lumpy, and DELLY, and tandem duplications were merged from Pindel and DELLY. Finally, we combined SVs from 66 individual pigs together and constructed the whole SV merged set of the Chinese pigs. The genotyped set included the SVs with precise breakpoint information determined by DELLY and with at least 90% of regions overlapped with the merged set reciprocally. The SV loci with low genotyped quality, or with a missing rate of more than 0.05, or were in homozygous fixed status in all 66 individuals were removed for further population analysis.

### Local assembly and precise breakpoint identification

For the SVs discovered by the five callers, we assembled contigs around their breakpoints and further mapped to the corresponding regions of the reference genome. The assembly procedure was conducted by the Velvet v1.2.10 [61] and TIGRA-SV v0.4.2 [62] tools. For TIGRA-SV, we assembled the extracted reads near the inferred breakpoints (± 500 bp) corresponding to the carrier samples. Regarding Velvet, soft-clipped reads within +/- 1 kb of the start and end positions were extracted for assembly. We aligned these SVs with breakpoints, as well as less than 60 kb, to the corresponding regions (1 kb for TIGRA-SV and 2 kb for Velvet) with AGE v0.4 [63]. Finally, we combined the results and utilized the alignments assembled by TIGRA-SV, which largely agreed with the result from Velvet. Breakseq v1.3 [28] was then used to infer the formation mechanisms of the recalibrated SVs.

### Population genetics analysis

PCA clustering and stratification analyses were performed by GCTA v1.24.7 [64] and Admixture Tools v1.23 [65], respectively. The calculation of variant frequency was conducted by VCFtools v0.1.12 [66]. LD pattern was calculated using PLINK2 [67] with options ‘—ld-window 99999—ld-window-kb 1000 -ld-window-r2 0.2’ to obtain all information in each 1 Mb window.

We used VariScan v2.0.3 [68] to compute Tajima’D for the deletions on each chromosome, with a sliding window for 20 deletions and a step size for 10 deletions. However, the further estimation of Tajima’D on chromosome X was performed by a 1 Mb window size. VCFtools was used for calculating Fst among the groups with the window size set to 1 Mb. We performed permutation test to access the significance for Fst by shuffling group labels, where group size in each group was kept the same, and re-calculated Fst statistics for 10,000 times. Under the null hypothesis of no differences between two groups, an empirical p value for the Fst was estimated as P = (n+1)/10,001, where n was the counts of the permutated sets for which the Fst was equal to or greater than the observed Fst in the real data. Re-sequencing data of the additional 26 European pigs were obtained from NCBI (https://www.ncbi.nlm.nih.gov/). The D value was applied to evaluate the measurement of differences:

$$D=\frac{{ơ}_{T}^{2}-\frac{{ơ}_{NCPG}^{2}n_{NCPG}+{ơ}_{SCPG}^{2}n_{SCPG}}{n_{NCPG}+n_{SCPG}}}{{ơ}_{T}^{2}}$$

where ${ơ}_{T}^{2}$, ${ơ}_{NCPG}^{2}$ and ${ơ}_{SCPG}^{2}$ stand for the whole Fst variances among SCPG and NCPG, Fst variances in NCPG and Fst variances in SCPG, respectively; and *n*_*NCPG*_ and *n*_*SCPG*_ denote the population size number of NCPG and SCPG, respectively.

### Functional analysis and plotting

The genomic information for transcripts was downloaded from ENSEMBL BioMart (http://www.ensembl.org/biomart). Gene Ontology (GO) enrichment analysis was performed by R package topGO [69]. Statistical tests and plotting were conducted using R v3.2.2 program, Circos v0.67 [70] and in-house Perl scripts. A permutation test in each analysis was performed by 10,000 runs.

## Supporting information

S1 Fig Size distribution of deletions in 66 Chinese pigs. (TIF)

S2 Fig Counts of different types of SVs distributed in SV hotspots. (TIF)

S3 Fig Heterozygosity of SNP variants in Chinese pigs. **T**he horizontal and vertical axes depict different Chinese pig breeds and heterozygosity normalized by log10 value, respectively. BMX, Bamaxiang; EHL, Erhualian; HT, Hetao; LAIWU, Laiwu; LC, Luchuan; MIN, Min; GST, Tibetan (Gansu); SCT, Tibetan (Sichuan); TIBT, Tibetan (Tibet); YNT, Tibetan (Yunnan); WZS, Wuzhishan; WILD, Southern Chinese wild boar.
(TIF)

S4 Fig **Heterozygosity of Chinese pigs denoted by inversions (A) and tandem duplications (B) in Chinese pigs.** The horizontal and vertical axes depict different Chinese pig breeds and heterozygote counts normalized by log10 value, respectively, and the abbreviations are the same as above.
(TIF)

S5 Fig **Principal component analysis based on inversions (A) and tandem duplications (B) of 66 Chinese pigs.** The abbreviations are the same as above.
(TIF)

S6 Fig Admixture stratification based on inversions among 12 breeds of Chinese pigs. The stratification analysis is shown in the case of K = 2, K = 3 and K = 4, and the abbreviations are the same as above.
(TIF)

S7 Fig Admixture stratification based on tandem duplications among 12 breeds of Chinese pigs. The stratification analysis is shown in the case of K = 2, K = 3 and K = 4, and the abbreviations are the same as above.
(TIF)

S8 Fig The sharing status of deletion alleles sharing across three pig groups. (TIF)

S9 Fig The sharing status of inversion alleles sharing across three pig groups. (TIF)

S10 Fig The sharing status of tandem duplication alleles sharing across three pig groups. (TIF)

S11 Fig Analysis of principal component (A), admixture stratification (B), and variants frequency distribution (C) performed with SNPs.
(TIF)

S12 Fig Chromosome wide Tajima’s D values in NCPG (A), SCPG (B), and TWPG (C) groups.
(TIF)

S13 Fig Tajima’s D values of SNPs on chromosome X in three groups of Chinese pigs. Gray and red regions represent continuous negative blocks, and the red rectangle area overlaps with the significantly negative region for deletions.
(TIF)

S14 Fig Locations of differentiated deletion sites in TPG vs. DPG (A) and SCPG vs. NCPG (B) groups.
(TIF)

S15 Fig Fst level of deletion sites distributed in INTRON, C50, and D50 groups in the comparison of deletions emerged in DEL-DC and not emerged in DEL-DC (A), and each breed of SCPG vs. WILD and each breed of NCPG vs. WILD (B).
(TIF)

S1 Table The merged set of SVs distributed in Chinese pigs. (XLSX)

S2 Table Genotyped set of SVs. (XLSX)

S3 Table Precise set of SVs. (XLSX)

S4 Table SV formation mechanisms. (XLSX)

S5 Table SV hotspot regions with formation mechanism proportions (Yellow: NHR dominant; Green: TEI dominant). (XLSX)

S6 Table Genes inside of the differentiated 12 Mb on X chromosome identified by the SCPG vs. NCPG comparison. (XLSX)

S7 Table Genes with missense SNPs and Fst > 0.8 within the differentiated 12 Mb region identified by the SCPG vs NCPG comparison. (XLSX)

S8 Table SRA accession number of European pigs analyzed in this study. (XLSX)

S9 Table Location of the top 5% of significant deletion sites of TPG vs. DPG. (XLSX)

S10 Table GO enrichment of genes that are identified in the TPG vs. DPG comparison to be potentially affected by the top 5% of significantly differentiated deletion sites. (XLSX)

S11 Table Location of the top 5% of significant deletion sites of SCPG vs. NCPG. (XLSX)

S12 Table Regions of DEL-DC clustered with high Fst levels of deletions and SNPs in high LD. (XLSX)

S13 Table Intronic and intergenic deletion sites compared to wild boars (Yellow: NCPG vs. WILD, Green: SCPG vs. WILD). (XLSX)

S14 Table GO enrichment of genes that are identified in the SCPG vs. NCPG comparison to be potentially affected by D50 group of deletions in DEL-DC. (XLSX)
