# Supplementary figures and images for: Genome-wide analysis of structural variants reveals genetic differences in Chinese pigs
Source: PLoS One. 2017 Oct 24;12(10):e0186721. doi: 10.1371/journal.pone.0186721 (PMC5655481; doi:10.1371/journal.pone.0186721)

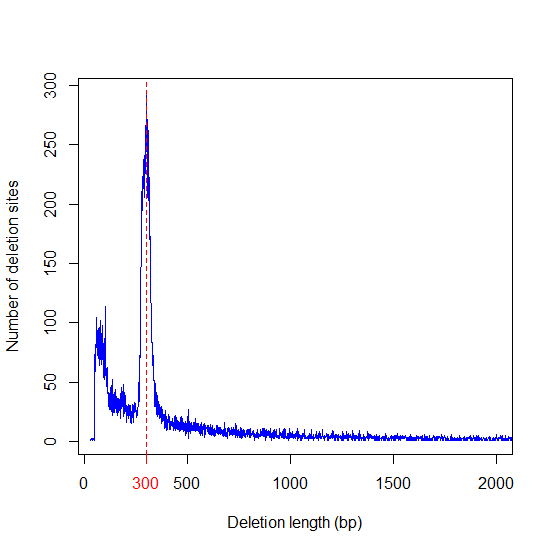

Supplement: S1 Fig — (TIF) [file pone.0186721.s001.tif]

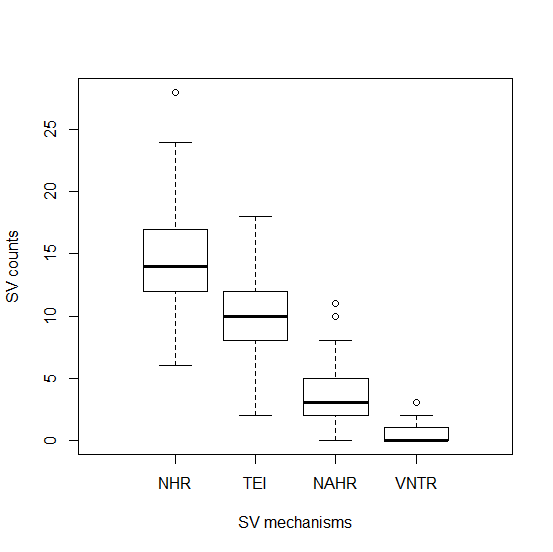

Supplement: S2 Fig — (TIF) [file pone.0186721.s002.tif]

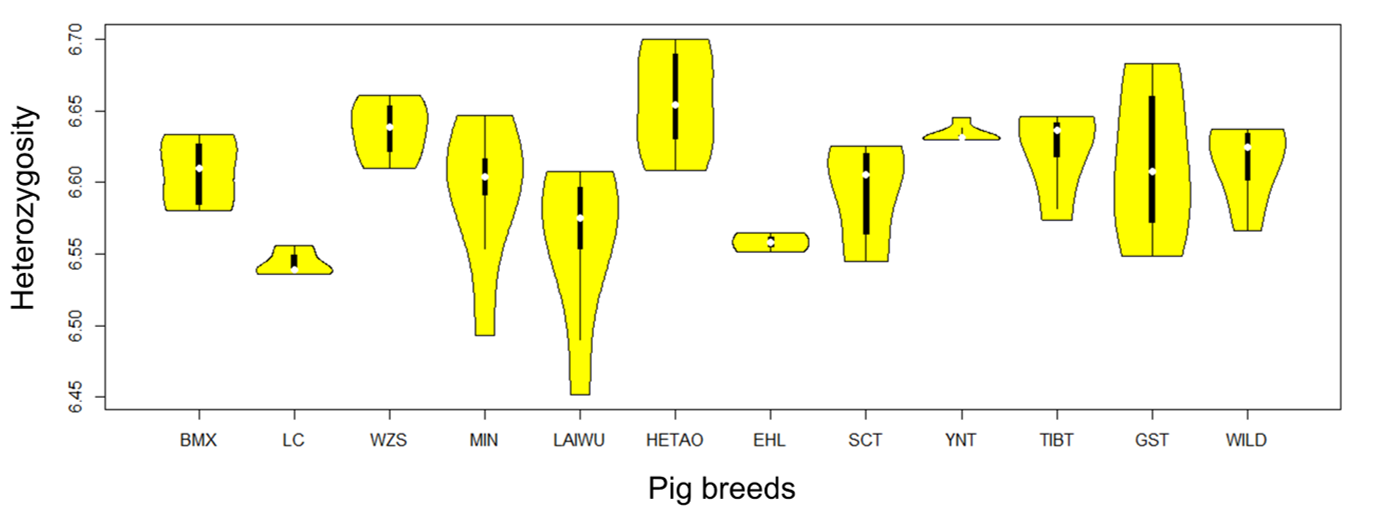

Supplement: S3 Fig — The horizontal and vertical axes depict different Chinese pig breeds and heterozygosity normalized by log10 value, respectively. BMX, Bamaxiang; EHL, Erhualian; HT, Hetao; LAIWU, Laiwu; LC, Luchuan; MIN, Min; GST, Tibetan (Gansu); SCT, Tibetan (Sichuan); TIBT, Tibetan (Tibet); YNT, Tibetan (Yunnan); WZS, Wuzhishan; WILD, Southern Chinese wild boar. (TIF) [file pone.0186721.s003.tif]

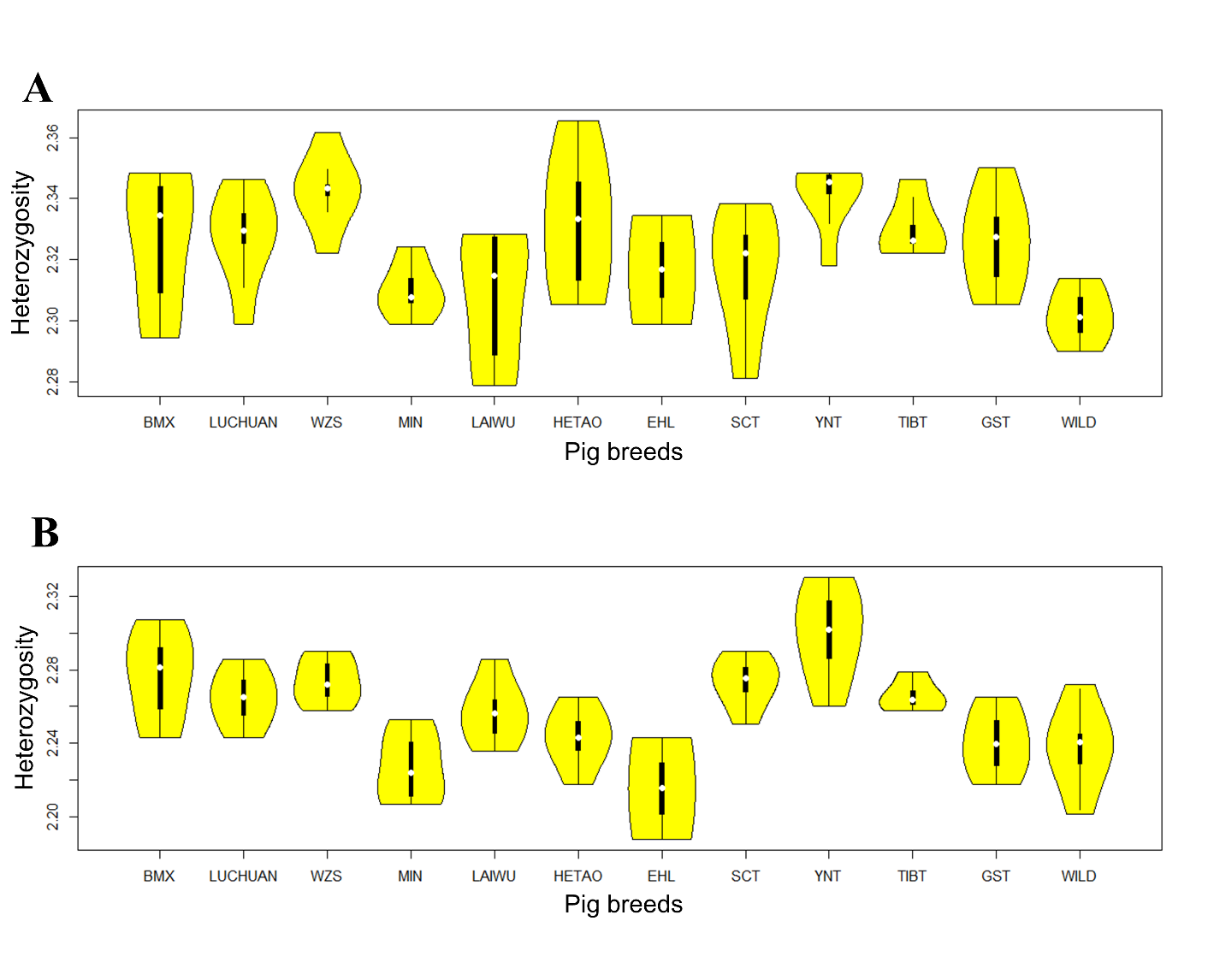

Supplement: S4 Fig — Heterozygosity of Chinese pigs denoted by inversions (A) and tandem duplications (B) in Chinese pigs. The horizontal and vertical axes depict different Chinese pig breeds and heterozygote counts normalized by log10 value, respectively, and the abbreviations are the same as above. (TIF) [file pone.0186721.s004.tif]

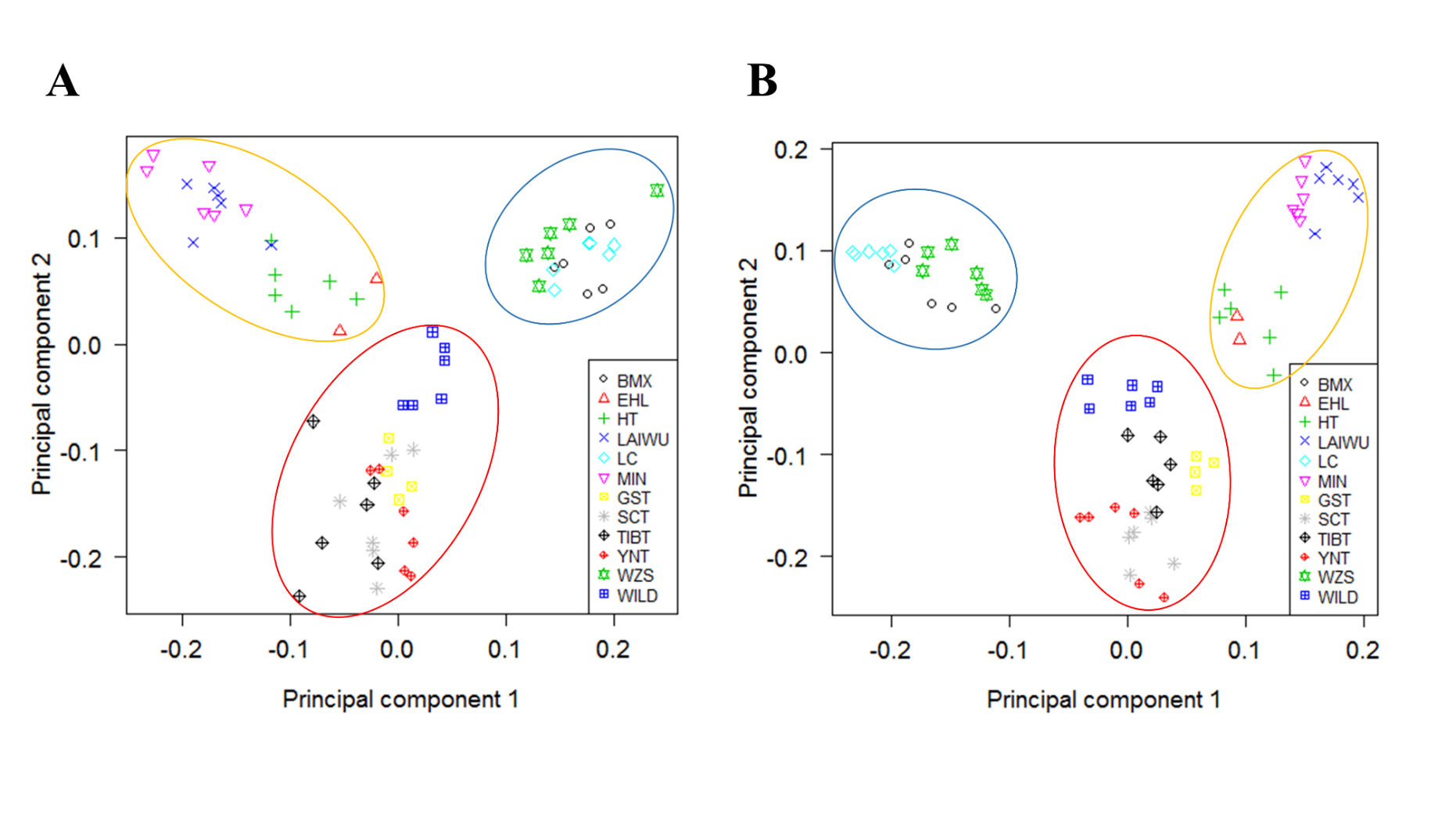

Supplement: S5 Fig — Principal component analysis based on inversions (A) and tandem duplications (B) of 66 Chinese pigs. The abbreviations are the same as above. (TIF) [file pone.0186721.s005.tif]

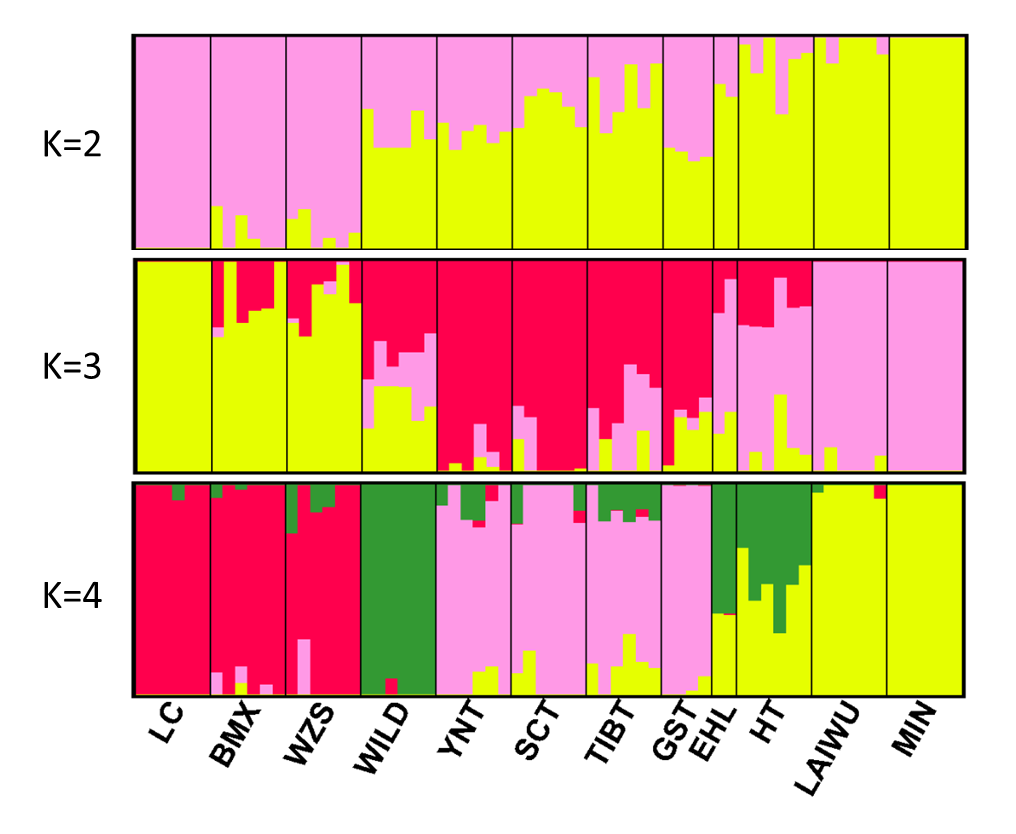

Supplement: S6 Fig — The stratification analysis is shown in the case of K = 2, K = 3 and K = 4, and the abbreviations are the same as above. (TIF) [file pone.0186721.s006.tif]

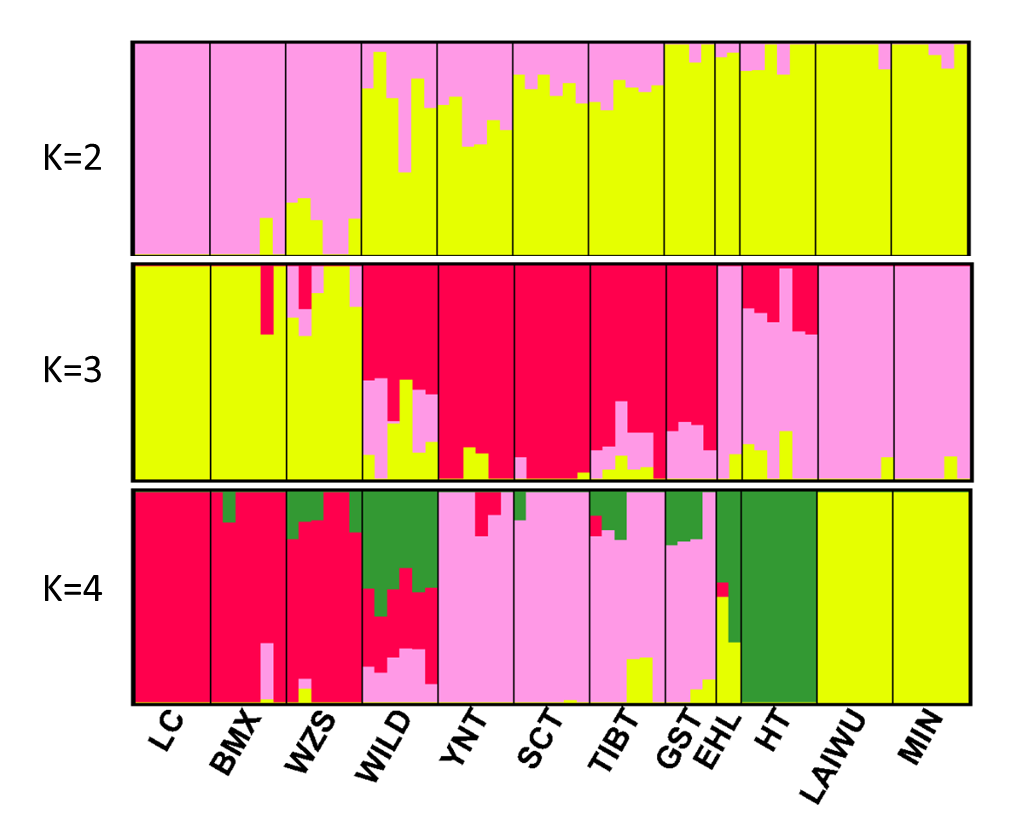

Supplement: S7 Fig — The stratification analysis is shown in the case of K = 2, K = 3 and K = 4, and the abbreviations are the same as above. (TIF) [file pone.0186721.s007.tif]

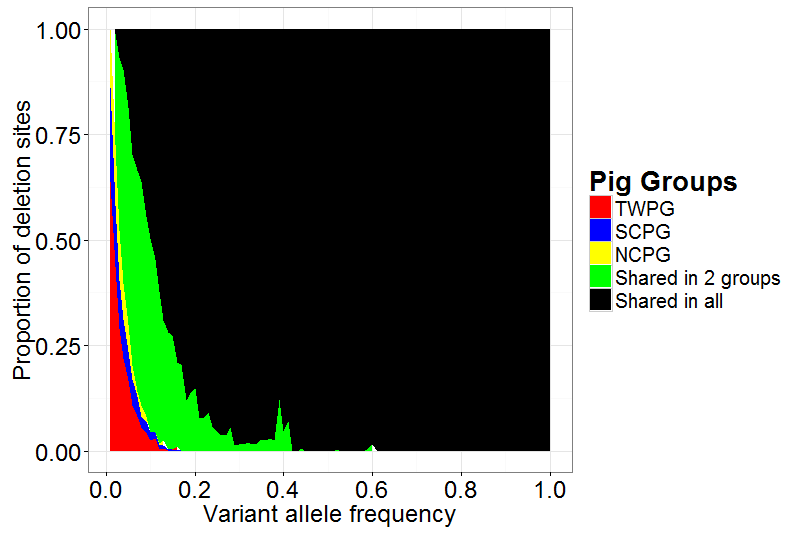

Supplement: S8 Fig — (TIF) [file pone.0186721.s008.tif]

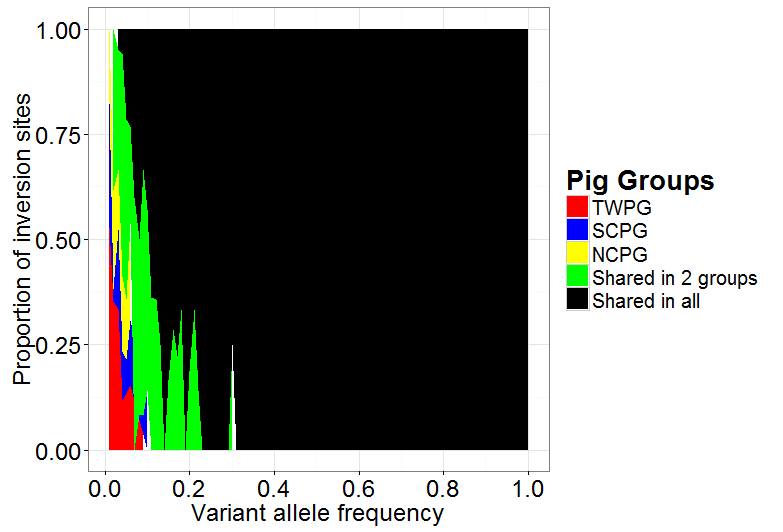

Supplement: S9 Fig — (TIF) [file pone.0186721.s009.tif]

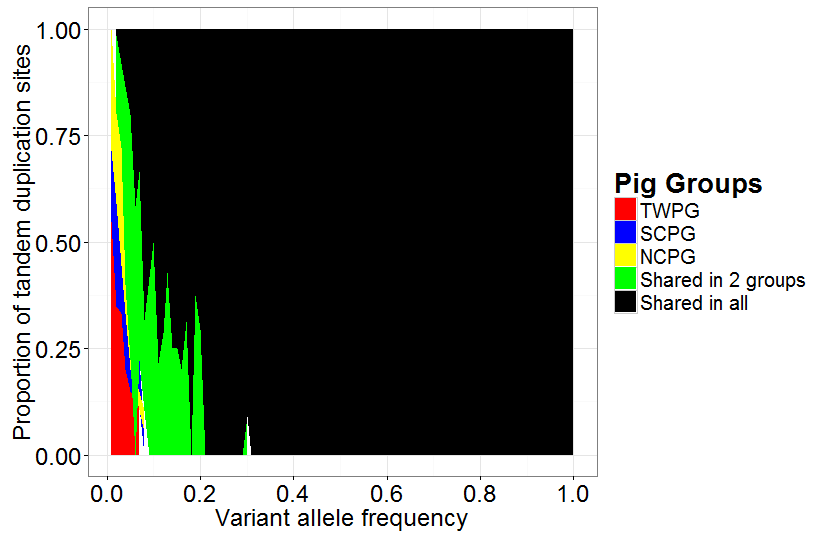

Supplement: S10 Fig — (TIF) [file pone.0186721.s010.tif]

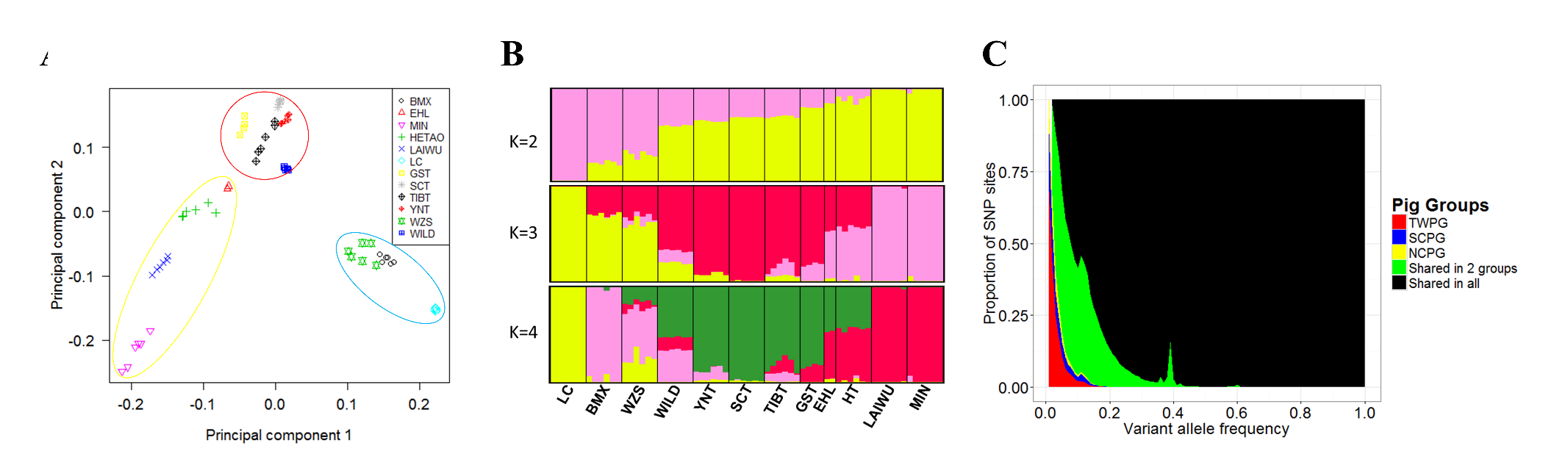

Supplement: S11 Fig — Analysis of principal component (A), admixture stratification (B), and variants frequency distribution (C) performed with SNPs. (TIF) [file pone.0186721.s011.tif]

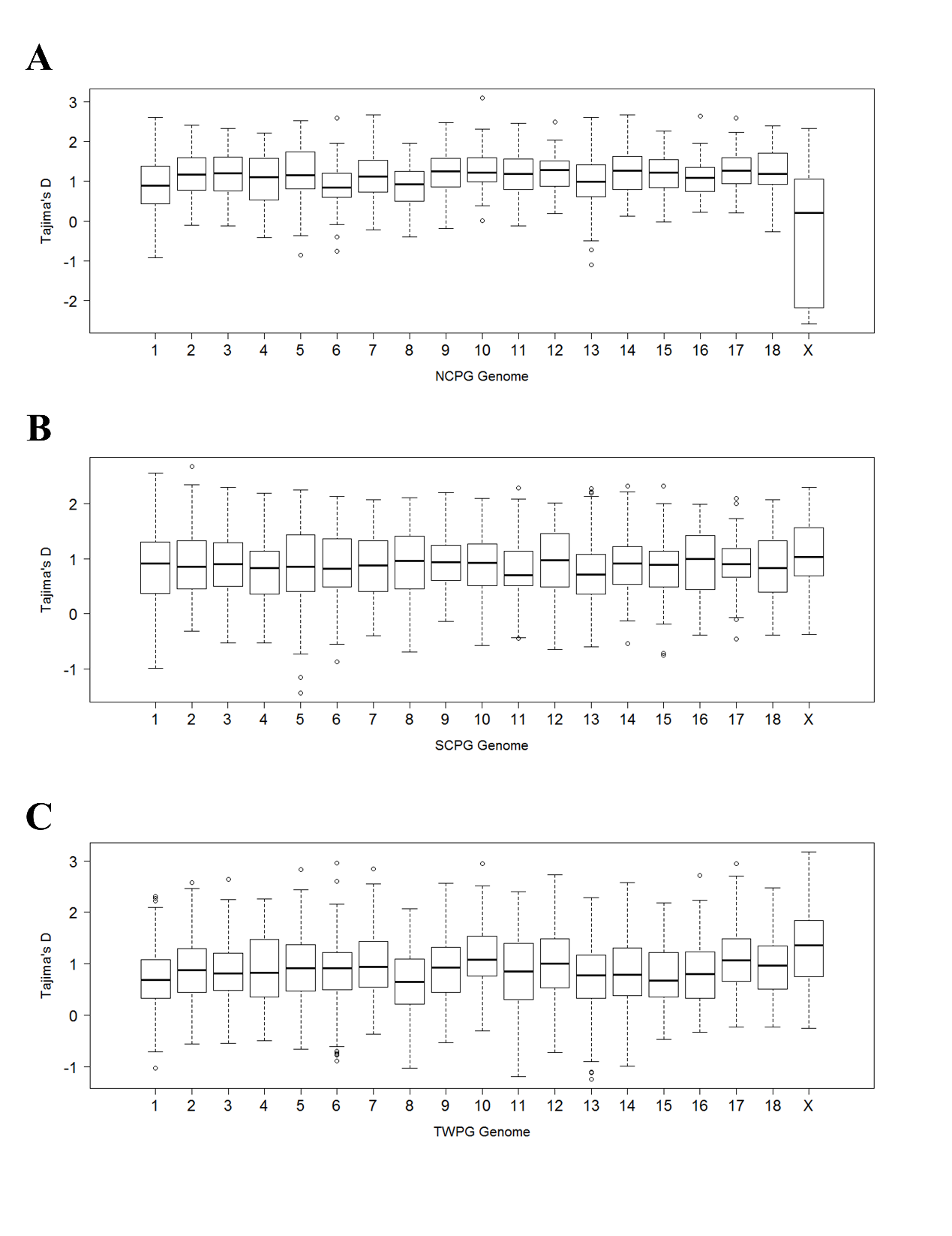

Supplement: S12 Fig — Chromosome wide Tajima’s D values in NCPG (A), SCPG (B), and TWPG (C) groups. (TIF) [file pone.0186721.s012.tif]

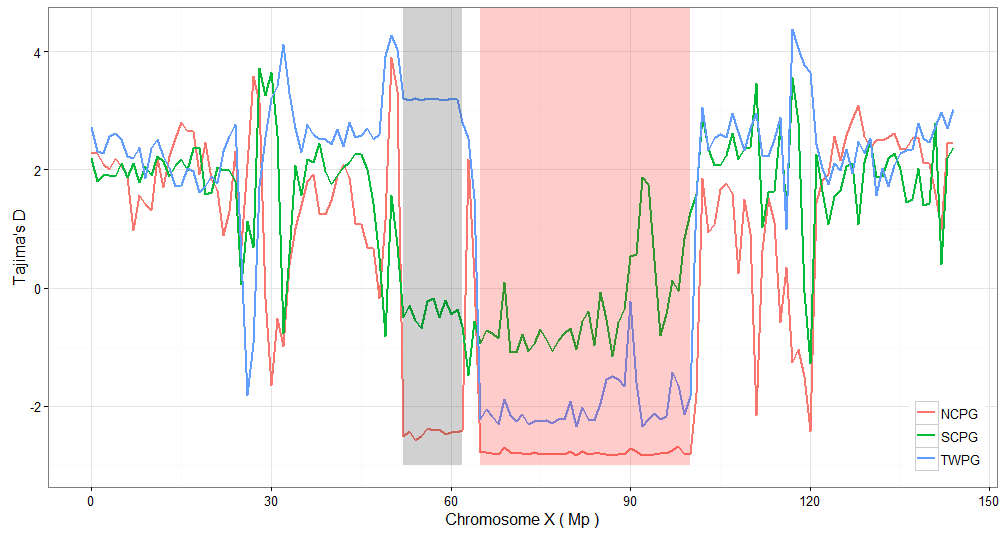

Supplement: S13 Fig — Gray and red regions represent continuous negative blocks, and the red rectangle area overlaps with the significantly negative region for deletions. (TIF) [file pone.0186721.s013.tif]

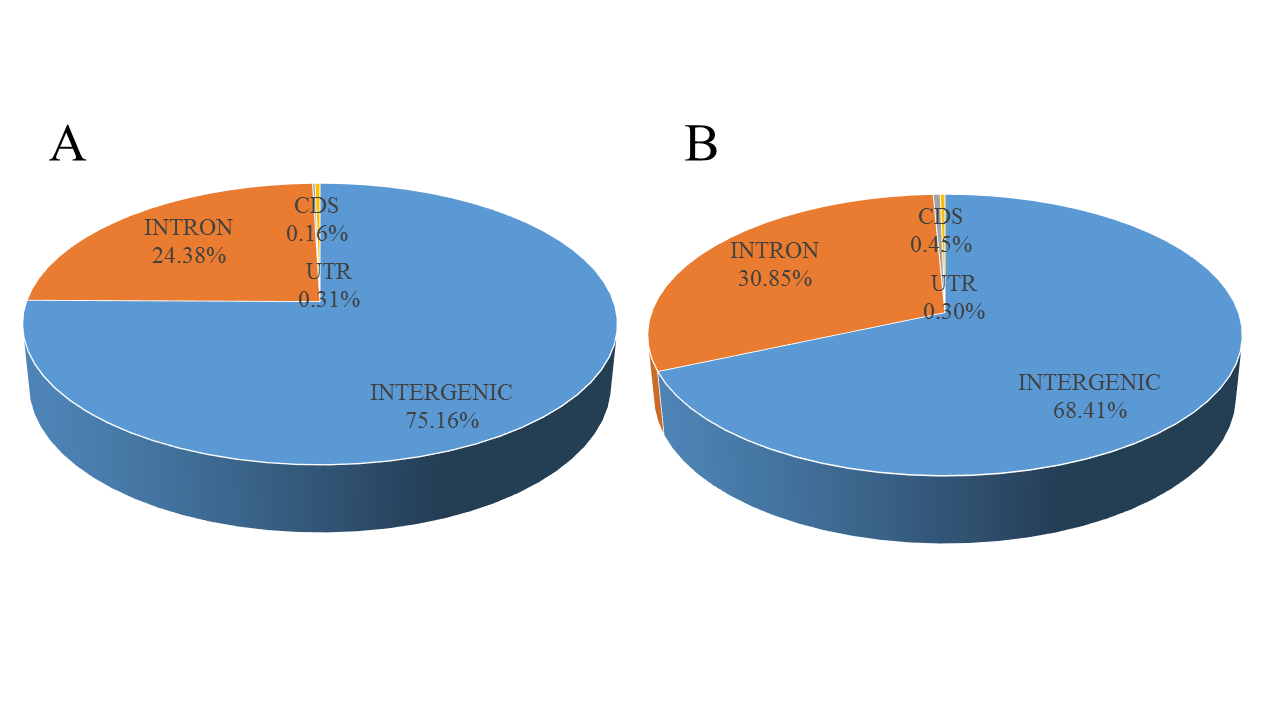

Supplement: S14 Fig — Locations of differentiated deletion sites in TPG vs. DPG (A) and SCPG vs. NCPG (B) groups. (TIF) [file pone.0186721.s014.tif]

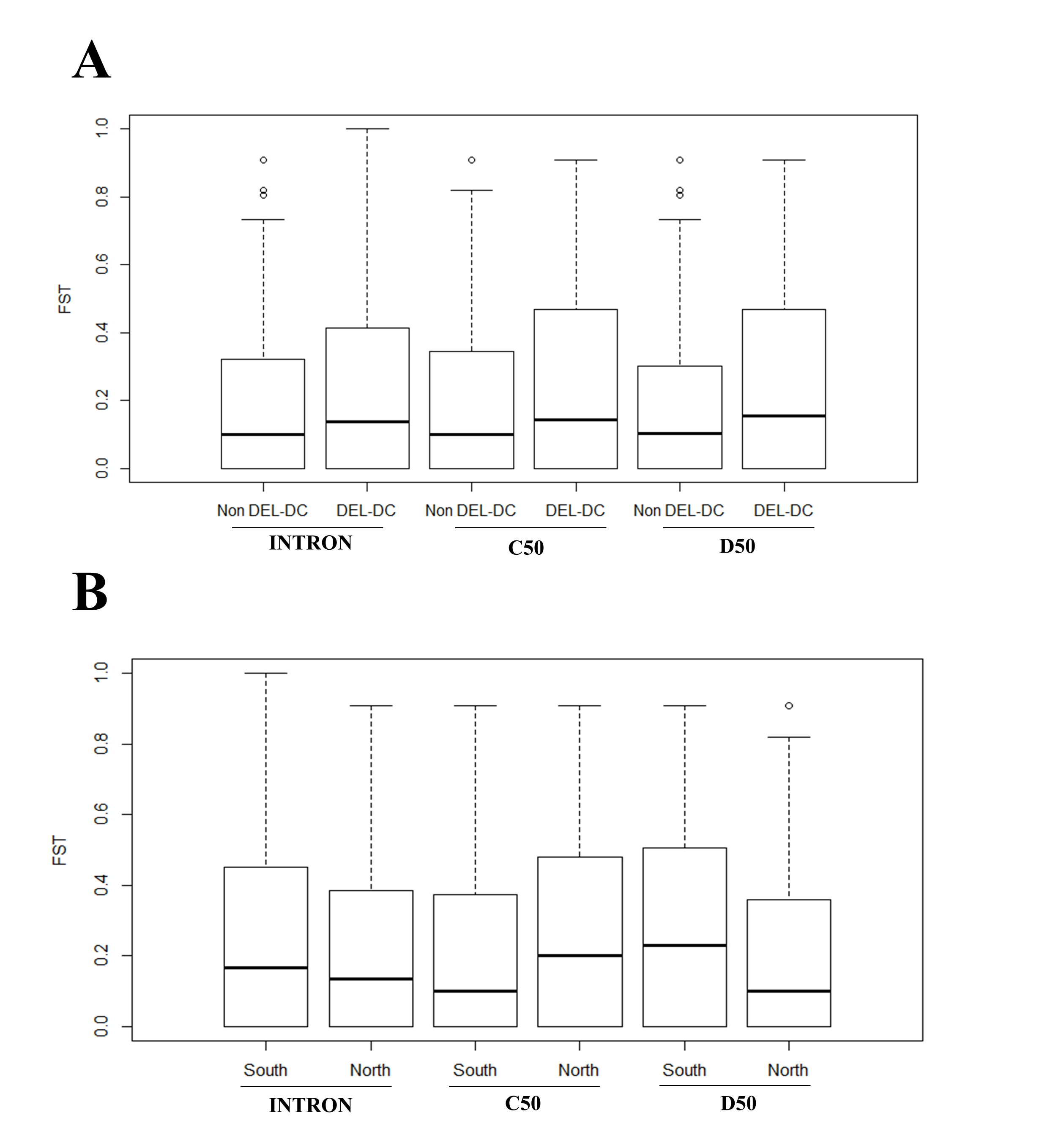

Supplement: S15 Fig — Fst level of deletion sites distributed in INTRON, C50, and D50 groups in the comparison of deletions emerged in DEL-DC and not emerged in DEL-DC (A), and each breed of SCPG vs. WILD and each breed of NCPG vs. WILD (B). (TIF) [file pone.0186721.s015.tif]
